# Phytohormone biosynthesis and transcriptional analyses provide insight into the main growth stage of male and female cones *Pinus koraiensis*

**DOI:** 10.3389/fpls.2023.1273409

**Published:** 2023-10-11

**Authors:** Yan Li, Minghui Zhao, Kewei Cai, Lin Liu, Rui Han, Xiaona Pei, Lina Zhang, Xiyang Zhao

**Affiliations:** ^1^ Jilin Provincial Key Laboratory of Tree and Grass Genetics and Breeding, College of Forestry and Grassland Science, Jilin Agricultural University, Changchun, China; ^2^ College of Life Science, Jilin Agricultural University, Changchun, China; ^3^ College of Horticulture, Jilin Agricultural University, Changchun, China; ^4^ School of Information Technology, Jilin Agricultural University, Changchun, China

**Keywords:** Pinus koraiensis, male and female cones, phenological, phytohormone, transcriptome

## Abstract

The cone is a crucial component of the whole life cycle of gymnosperm and an organ for sexual reproduction of gymnosperms. In *Pinus koraiensis*, the quantity and development process of male and female cones directly influence seed production, which in turn influences the tree’s economic value. There are, however, due to the lack of genetic information and genomic data, the morphological development and molecular mechanism of female and male cones of *P. koraiensis* have not been analyzed. Long-term phenological observations were used in this study to document the main process of the growth of both male and female cones. Transcriptome sequencing and endogenous hormone levels at three critical developmental stages were then analyzed to identify the regulatory networks that control these stages of cones development. The most significant plant hormones influencing male and female cones growth were discovered to be gibberellin and brassinosteroids, according to measurements of endogenous hormone content. Additionally, transcriptome sequencing allowed the identification of 71,097 and 31,195 DEGs in male and female cones. The synthesis and control of plant hormones during cones growth were discovered via enrichment analysis of key enrichment pathways. *FT* and other flowering-related genes were discovered in the coexpression network of flower growth development, which contributed to the growth development of male and female cones of *P. koraiensis*. The findings of this work offer a cutting-edge foundation for understanding reproductive biology and the molecular mechanisms that control the growth development of male and female cones in *P. koraiensis*.

## Introduction

Ecological and economic value may be found in the monoecious plant *Pinus koraiensis*, an evergreen conifer of Pinaceae with a tree height of 40 m and a breast height diameter of 1 m (Li et al., 2022; Park et al., 2016; Ren et al., 2022). *P. koraiensis* is mostly distributed in China’s Changbai Mountains, Laoyeling Mountains, Zhangguangcailing Mountains, Xiaoxinganling Mountains and Wanda Mountains, as well as in Japan, the Far East region of Russia and the Korean Peninsula (Aizawa et al., 2012; Shi et al., 2020; Wei et al., 2022; Zhang et al., 2022). It has state approval to be designated as a secondary wild plant for national protection (Li et al., 2020). Due to its plentiful lipid, amino acids, proteins, and other nutrients that can successfully decrease blood lipids and cholesterol in humans, *P. koraiensis* seed kernels have become one of the most significant nut foods accessible (Song et al., 2021; Ryuk et al., 2022; Wang et al., 2022). It frequently takes the natural *P. koraiensis* 20 to 30 years to produce cones due to its slow growth cycle. And its inconsistent blooming time and low yield during cone setting result in an exceedingly unpredictable seed production, which results in farmers suffering huge financial losses (Li et al., 2021; Sun et al., 2021; Wu et al., 2022; Wang et al., 2021a). Here, *P. koraiensis* seed yield is mostly influenced by the number and growth development of male and female cones. There has not been any information published about the morphologic change, hormone levels, or gene expression that occurs while *P. koraiensis* male and female cones develop. Therefore, it is crucial to investigate the growth developmental characteristics and mechanism of this species, as this knowledge may help to increase *P. koraiensis* seed production.

Plants go through several developmental stages during their life cycle, but the formation of flower is the most significant stage since it signifies the critical transition from vegetative to reproductive growth (Wellmer and Riechmann, 2010; Liu et al., 2016; Wang et al., 2021b). The development of flowers is regulated by both the external environment and their internal genes, which have long built a sophisticated and precise gigantic regulatory network (Mouradov et al., 2002; Ó’Maoiléidigh et al., 2014; Chen et al., 2018). Numerous elements, including photoperiod, temperature, phytohormones, vernalization, autonomy, and gene regulation, have been demonstrated to mediate the flowering process in plants with the advent of molecular biology and genetics (Cao et al., 2021). However, it has been suggested that phytohormones hold the key to controlling floral sex differentiation and development (Cheng and Zhao, 2007). The impact of exogenous hormones on *Castanea henryi* flowers development was discussed by Fan et al. The findings demonstrated that gibberellins and abscisic acid were mostly deposited in male flowers, while cytokinins were significantly more abundant in female flowers than in male flowers, demonstrating the dynamic changes in hormones throughout floral development (Fan et al., 2017). Transcriptome and hormone analysis of major buds in *Crocus sativus* L. revealed that GA_4_ content was a prerequisite for flower induction and development (Renau-Morata et al., 2021). Additionally, research has demonstrated that jasmonate is essential for reproductive activities such as plant male fertility and sex differentiation (Yuan and Zhang, 2015), and which mediates plant resistance to against necrotrophy (Zheng et al., 2022). Moreover, through direct ABA biosynthesis promotion and callose synthetase gene *CALLOSE SYNTHETASE 1* activation, the poplar SVL protein improves terminal bud endodormancy (Gao et al., 2021). Furthermore, many reports using angiosperms as model plants provide only limited references to gymnosperms. In the study of *Pinus radiata*, it was mentioned that the gibberellin complex (GA1, GA3, GA4, GA7, GA9) were closely related to the development of cone buds, which was mainly manifested in the significant differences in the content of different development stages (Ross et al., 1984). ABA has a complex regulatory mechanism for plant growth and development. When the ABA metabolic pathway is activated and the content decreases, the dormancy period of male cones of *Pinus tabulaeformis* is shortened and the growth of stems is accelerated (Zhang et al., 2022).

Genes have the ability to regulate the phenotypic characteristics of plants. Previous studies have identified the role and molecular mechanism of some key regulatory genes in plant flower growth. By triggering FT activity, *CO* controls the synthesis and transmission of flowering signals in the model plant *Arabidopsis thaliana* (An et al., 2004). FOREVER YOUNG FLOWER 1 (FYF1) and FYF2 are activators that, when combined, promote flower senescence and block flower abscission, respectively (Chen et al., 2022). The *SOC1* gene also plays a key role in the flower induction phase of *Persian walnut* flower growth, and the FT gene is strongly expressed at the flowering stage (Hassankhah et al., 2020). In addition, the researchers also concluded that TFL2, CO, NF-YC1 and NF-YC4 have a positive regulatory role in the formation of cones by identifying the function of gene families in male cones of *P. tabulaeformis* (Guo, 2020). A set of transcription factors known as the MADS-box gene family particularly recognizes and binds to distinctive DNA sequences. It controls a variety of gymnosperms and angiosperms plant development processes, such as the growth of flowers/cones, fruits, leaves, and roots (Taylor et al., 2002; Koshimizu et al., 2018; Susanne et al., 2019; Niu et al., 2022). At the bud stage and flower development stage of the chickpea, one study found that a total of 18 MADS-box genes were discovered, and the expression differential was the most significant of all transcription factors (Singh et al., 2013). *A. thaliana* contains 109 MADS transcription factor family. According to research, MADS domain proteins are present at different phases of plant flower development, and other transcription factor families work in conjunction with the MADS-box gene family to support the formation and development of flower organs (Smaczniak et al., 2012). In conifers, MADS gene family are thought to be a regulatory mediator for the transition from juvenile to adult (Carlsbecker et al., 2004; Carlsbecker et al., 2013). For example, Ma et al. focused on the functional characteristics of the MADS gene family by performing time-dynamic transcriptome analysis on *P. tabulaeformis*. The results showed that it was not only closely related to aging, but also played a key role in the transition from vegetative growth to reproductive growth of *P. tabulaeformis* (Ma et al., 2021). Similar conclusions were also confirmed in *Picea abies* (Akhter et al., 2018).

In the field of conifers development biology, male and female cone development has long been a focus of investigation. Improved seed breeding, introduction, high yield, and steady cultivation can all be aided by understanding how plants make cones (Goslin et al., 2017; Zheng and Xia, 2022). High-performance liquid chromatography and transcriptome sequencing methods were employed in this study to examine the changes in hormone and gene levels at various stages of growth in *P. koraiensis* male and female cones. We started by performing morphological observations on the main growth developmental stage after the formation of male and female cones. Second, the amounts of eight plant hormones that are produced by the body were examined in cones at three significant growth phases. Finally, gene-specific alterations related to three significant developmental stages were examined using transcriptome sequencing. Through systematic phenological observation, hormone determination, and gene level analysis, this study seeks to better understand the relationship between male and female cones development and hormone content and genes. It also provides a useful reference for *P. koraiensis* genetic engineering breeding.

## Materials and methods

### Experimental material and cone flower morphology observation

*P. koraiensis* is mainly distributed in Northeast China and has unique reproductive biology. The plant material used in this study was grown in the *P. koraiensis* seed orchard of the Linjiang Forestry Bureau, Jilin Province, China (41°05’N, 126°06’), with a tree age of 30 years. In this study, the male and female cones morphology of *P. koraiensis* plants was observed from the middle of April through late June on a single plant that was free of disease and insect pests. The experimental samples from the three typical growth phases of the male (MS1, MS2 and MS3) and female (FS1, FS2 and FS3) cones were gathered, including bud burst, sheath dehiscence stage and maturation stage. In each stage, a mixed sample of six male or female cones with the same growth developmental stage was chosen, and three biological replicates were set up for each stage sample. After removing the sheathings, all samples were promptly frozen in liquid nitrogen to prevent RNA degradation. They were subsequently stored at -80°C for RNA extraction, database construction, and transcriptome sequencing.

### Measurement of hormone content

To explore the relationship between male and female cones growth development and endogenous hormones, high-performance liquid chromatography (HPLC) and enzyme-linked immunosorbent assay (ELISA) were used by the Shanghai Enzymatic Biotechnology Company Ltd. (Shaihai, China) to measure the concentrations and dynamic changes of eight endogenous hormones during three typical stages of cone flowers growth. These hormones included gibberellins (GA), jasmonic acid (JA), auxin (IAA), abscisic acid (ABA), cytokinin (CTK), ethylene (ETH), brassinosteroids (BR) and zeatin (ZT). Using IBM SPSS 26 software, differences were examined for all hormone content data, and multiple comparison tests were run using the Student-Newman-Keuls (S-N-K) function. The different letters in the bar chart indicate significant differences (*P* < 0.05).

### RNA extraction and transcriptome sequencing

Total RNA was isolated using TRIzol reagent (Invitrogen), and then the concentration and purity were assessed using the Agilent Bioanalyzer 2100 system. High-quality RNA was utilized to create cDNA libraries, which were subsequently sequenced on an Illumina HiSeq platform (Illumina, San Diego, CA, USA) utilizing a combination probe anchored polymeric method and 150 bp long paired-end reads to generate raw data for further analysis. The National Center for Biotechnology Information’s (NCBI) SRA database contains transcriptome sequencing data with the accession number PRJNA903230. Strict quality control is used on the data to make sure it is of a sufficiently high enough standard. The raw data were cleaned of adaptor sequences and poor-quality bases using the Fastp software (version 0.12). At the same time, the GC content was extracted, and clean reads for subsequent analysis were obtained. For projects without reference genomes, clean reads need to be spliced to obtain reference sequences for subsequent analysis. *De novo* assembly of unigenes was performed using Trinity software (v2.11.0), and the transcriptome created by splicing clean reads on the Trinity server was used as the reference sequence. Finally, each sample’s clean reads were mapped to the reference sequence. This process used bowtie2 in software RSEM. DESeq2 was used to perform differential expression analysis between comparison groups. Benjamini-Hochberg was used to correct for the *P* value and obtain the false discovery rate (FDR) (Chen et al., 2020). The screening conditions of differential genes were ∣log2Fold Change∣≥1 and FDR < 0.05 (Love et al., 2014). The software BLAST was used to match the Unigene sequence with KEGG and GO in order to determine functional properties. HMMER software was used to match the predicted amino acid sequence of Unigene with the Pfam database to obtain the annotation data for Unigene. Using the ClusterProfile R package, graphical analysis of the enrichment findings was carried out (Young et al., 2010; Liao et al., 2014). Using the STRIN protein interaction database (http://string-db.org), homologous proteins shared by male/female cones and *A. thaliana* were found to obtain the interaction network between male and female cone candidate proteins. The final protein interaction regulatory network was then created by entering the protein interaction results into Cytoscape software. It should be highlighted that TBtools software was used to create and display each heatmap used in this study (Chen et al., 2020).

### Real-time quantitative PCR analysis

Using the 2^-ΔΔCT^ method and quantitative real-time polymerase chain reaction (qRT-PCR), the transcript abundance of 8 genes that are differentially expressed during the growth development of male and female cones was chosen and quantified. Each reaction was set to three technical replicates. The ABI 7500 RT PCR system was used for the qRT-PCR procedure. Using the online tool (https://sg.idtdna.com/scitools/Applications/RealTimePCR/default.aspx) and using 18S-RNA as the reference gene, all primers for this study were created. In **Table S1**, the qRT-PCR programs are displayed.

## Results

### Changes in main phenology during the male and female cones growth of *P. koraiensis*

**Figure 1:**
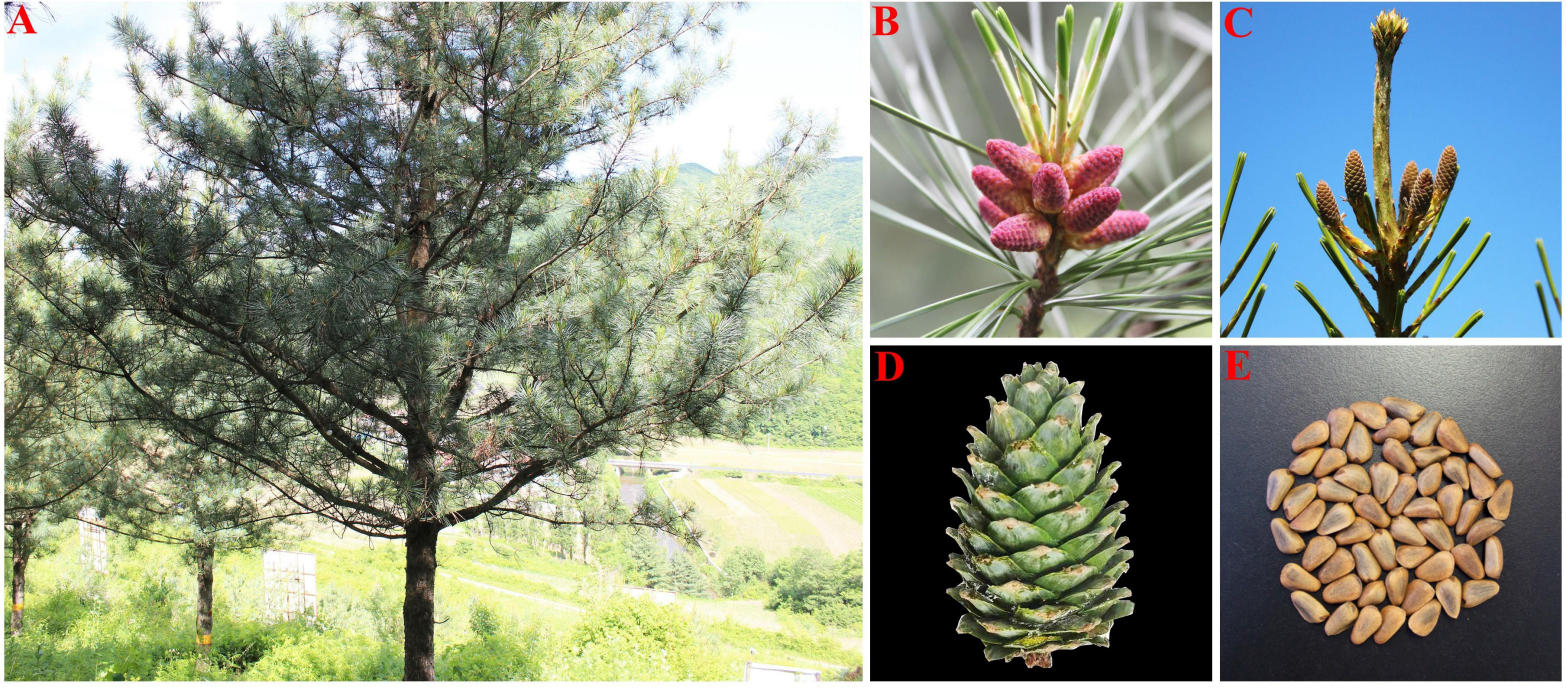
Morphological characteristics of *P. koraiensis*. [**(A)** single plant; **(B)** male cone; **(C)** female cone; **(D)** cone; **(E)** seed].

*P. koraiensis* is an evergreen monoecious conifer species with unisexual cones that are primarily pollinated by the wind. The tree has long branches, an umbrella-shaped crown, and black-gray bark that grows to a height of 40 meters. It is a sporophyte and lacks real, full blooms. The male, known as a microspore or male cone, has a form resembling a wheat grain and is typically born near the base of lateral branches and new branch tips. Megaspore, or female cone with pineapple-shaped petals, are female cones that develop at the top of the canopy beneath the terminal buds of fresh growth. Female cones of *P. koraiensis* continue to grow after being pollinated by male cones until the seed cones are ready the following autumn. When the cone reaches maturity, the ovuliferous scale spreads outward, exposing the seeds that had been concealed behind it (**Figure 1**).

**Figure 2:**
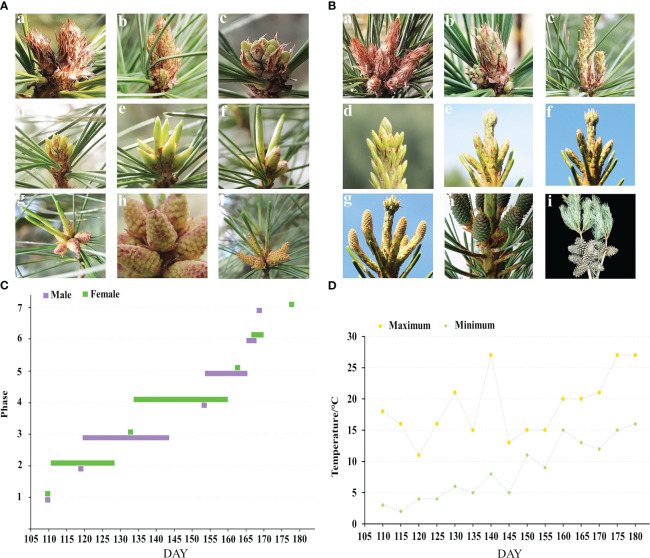
Phenological change in the male and female cones flowering process of *P. koraiensis*. **(A)** represents male cones, and **(B)** represents female cones. In **(A)**, (a) describes buds in winter, (b) phase 1: budding state, (c) phase 2: formation of male cone, (d, e) phase 3: elongation of male cone, (f) phase 4: formation of anther, (g) phase 5: mature anther, (h) phase 6: pollen dispersal from pollen sac, and (i) phase 7: withering of male cone. In **(B)**, (a) describes buds in winter, (b) phase 1: budding state, (c) phase 2: growing stage of terminal buds, (d) phase 3: bud splaying and formation of female cones, (e) phase 4: elongation of female cones, (f) phase 5: female cone breaking, (g) phase 6: female cone pollination, (h) phase 7: cone setting, and (i) describes mature cones. **(C)** Growth period of male and female cones, and **(D)** temperature change in the external environment during the growth period.

There are seven phases in the morphological growth of the male and female cones of *P. koraiensis* (**Figure 2C**). The male and female cone buds have rust yellow coloring and pilose surfaces with hairs when they are dormant in the winter. The sap starts to flow when the outside temperature reaches 0 to 5°C (**Figure 2D**). In the early stages of the growth of male cones, as seen in **Figure 2A**, it is challenging to identify male cone buds from leaf buds with a length of 2.45-4.64 mm (c, d). The male cone buds gradually grew coarse, elliptic, and 5-7 mm in length as the temperature increased steadily, but the leaf buds continued to elongate. Male cones cannot yet be seen in their complete shape because they are still covered in sheaths. The sheath tears between 155 and 160 DAY, exposing the male cone head, which starts to turn from green to yellow or red with a length of 6.08-6.99 mm (f). The sheath and all male cones become visible when the pollen of those cones is ripe; at this point, it turns yellow or red with a length of 7.14-10.61 mm (h and **Figure 1B**). After 3 to 5 days of loose powder, the male cones start to droop and eventually shed their loose pollen (i). **Figure 2B** shows the female cone buds of *P. koraiensis* growing at the top of the trunk; at this time in growth, it is impossible to distinguish the female cone buds from the leaf buds (c). The growth point continues to grow upward as the temperature rises, and a gap gradually develops between the developing point and the cone bud. At this point, the female cone bud differs slightly from the leaf bud in that it thickens with a length of 4 to 6 mm (d). The sheath of female cones cracked during the last stages of growth, and the color of the female cones’ heads progressively changed from green to yellow or purple red with a length of 9.52-14.09 mm (e, f). The female cones have a pineapple form when the sheath is fully expanded, and the scales gradually unfold to allow pollination with a length of 22.86-24.71 mm (g). After effective pollination, the female cone continues to develop into a cone (h), and in the second year of the long season, ovules start to form on the inner side of the nucellus.

### Changes in phytohormones during the main growth process of cone flowers

**Figure 3:**
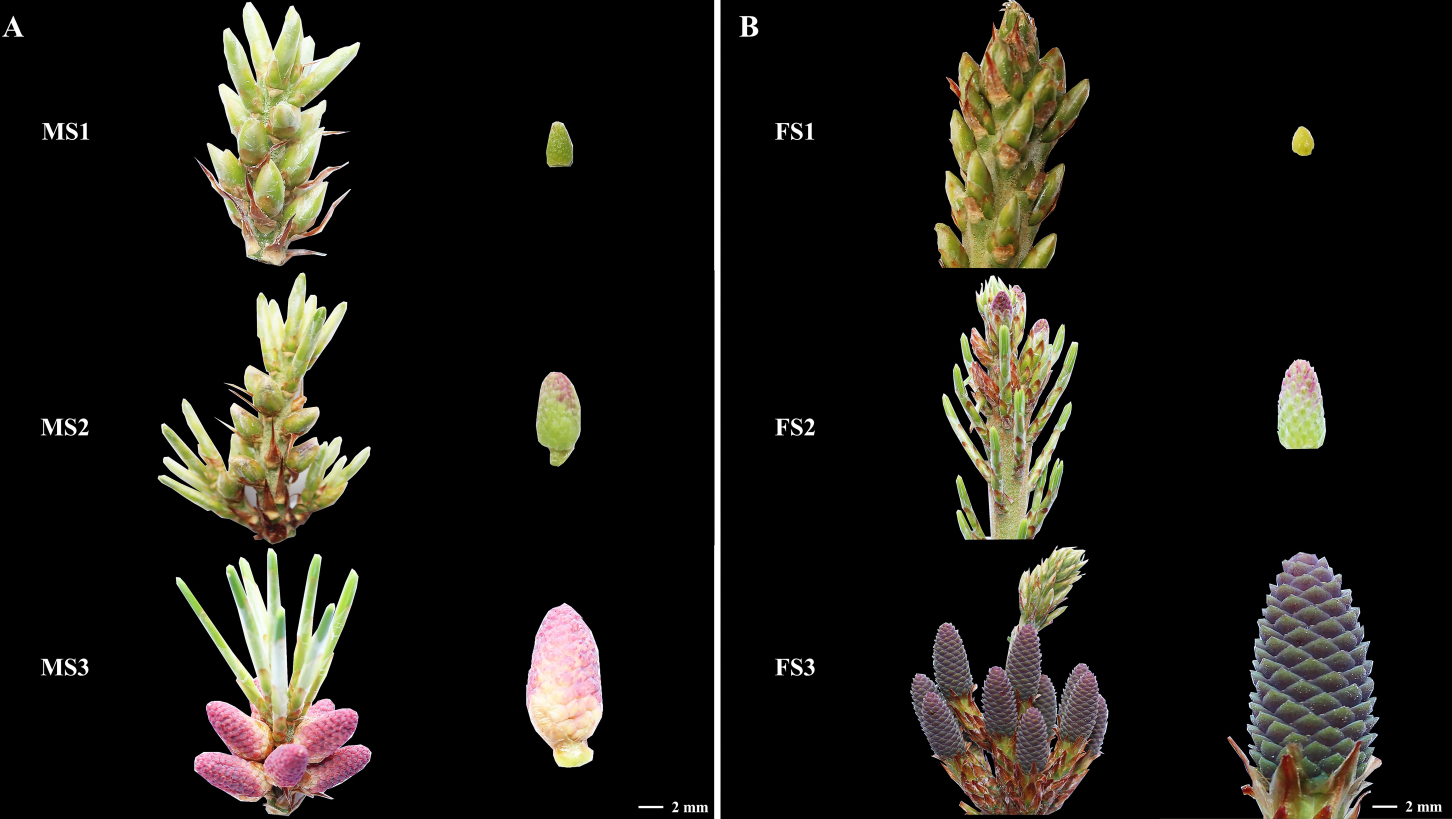
Plant samples. **(A)** the three crucial growth stages of male cones (MS1, MS2 and MS3); **(B)** the three critical growth stages of female cones (FS1, FS2 and FS3).

**Figure 4:**
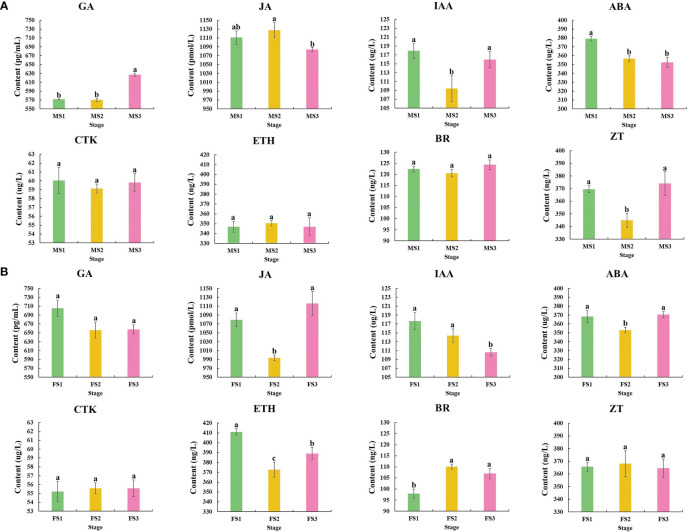
Changes in phytohormones during three crucial male and female cones growth stages. **(A)** Male cone; **(B)** female cone. The х-axis and y-axis indicate three crucial growth stages and phytohormone content of male and female cone, respectively. The IBM SPSS Statistics v26.0 software with the Student-Newman-Keuls multiple range test was used for the differences analysis, error bars show the SD of the means at n=3. Bars with different lowercase letters indicate significantly different (*P*<0.05).

Samples from the three stage were chosen to detect and analyze the content of these three crucial growth stages to investigate the changes in endogenous hormone content during the growth of male and female cone of *P. koraiensis* (**Figure 3**). In *P. koraiensis* male cone, the GA content increased significantly between the MS2 and MS3 stages, but not between the MS1 and MS2 stages. On the other hand, the ABA content increased early in growth and thereafter dropped. From MS1 to MS3, the contents of IAA and ZT declined first and then increased, whereas the contents of JA grew initially and then decreased. In particular, there were no appreciable variations between MS1 and MS3 in the CTK, ETH, or BR content (**Figure 4A**). Three phases of female cone growth showed significantly varying ETH contents (*P* < 0.05). The BR content greatly increased from FS1 to FS2; however, there was no discernible difference between FS2 and FS3. However, from FS1 to FS3, the IAA content gradually dropped. From FS1 to FS3, the contents of JA and ABA first declined and subsequently increased, but GA, CTK, and ZT exhibited no discernible change (**Figure 4B**).

### Statistics and functional enrichment of DEGs during the growth of male cone

**Figure 5:**
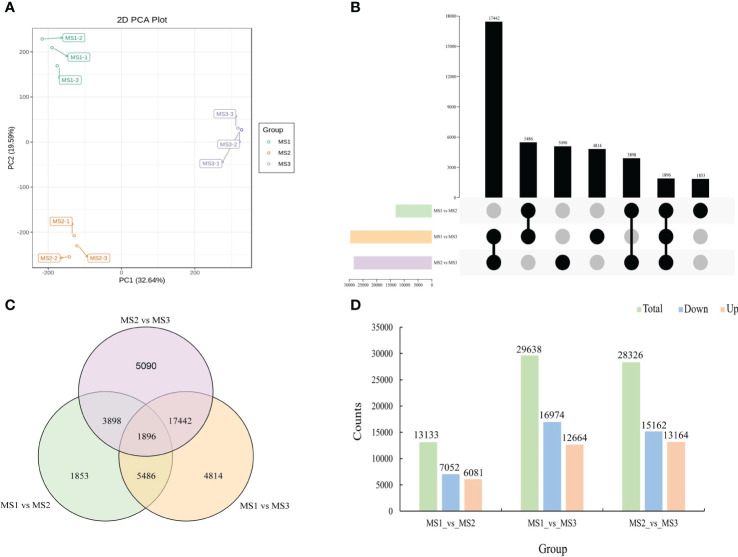
Identification of differentially expressed genes at the growth stages within male cone. **(A)** Principal component analysis (PCA) within samples; **(B)** UpSet plot of DEGs in MS1 vs. MS2, MS1 vs. MS3 and MS2 vs. MS3; **(C)** Venn diagram of DEGs in MS1 vs. MS2, MS1 vs. MS3 and MS2 vs. MS3; **(D)** comparative analysis of upregulated and downregulated DEGs between the three comparison groups.

Using the three critical stages of male cone growth shown in **Figure 3A**, RNA-seq was carried out to evaluate the transcriptional regulatory mechanisms driving the growth of male cone. A total of 503,243,650 raw reads were produced. A total of 477,865,358 clean reads were acquired after filtering and error correction for subsequent bioinformatics analysis. Additionally, the average Q20, Q30, and GC contents were 97.46%, 92.94%, and 44.58%, respectively, showing outstanding sequencing quality. Additionally, each sample’s clean reads were mapped to the reference sequence, and the percentage of reads that were mapped ranged from 85.08% to 86.04%, demonstrating the dependability of the RNA-seq data (**Table S2**). DEseq2 was employed to identify DEGs from each group to assess the relative gene expression level during the growth of the male cone (Chen et al., 2011). According to the PCA results, each stage’s sample replication was good, and there were significant variations between each stage (**Figure 5A**). A total of 71,097 DEGs were found among the genes involved in the development of male cone. Among these, 13,133 DEGs (6081 upregulated and 7052 downregulated) were found between MS1 and MS2, 29,638 DEGs (12,664 upregulated and 16,974 downregulated) between MS1 and MS3, and 28,326 DEGs (13,164 upregulated and 15,162 downregulated) between MS2 and MS3 (**Figures 5B-D**).

Additionally, as shown in **Table S3**, we were able to determine Unigene annotation information by matching Unigene sequences with a number of major databases. In the GO and KEGG databases, 54.21% and 45.12% of genes associated with male cone development were annotated, respectively. Functional enrichment analyses were carried out to compare the functionality of the DEGs found in the three groups. KEGG pathway analyses of DEGs among the three groups are compared in **Figure S1(A-C)**. In MS1 vs. MS2, the top four pathways were plant hormone signal transduction (ko04075), spliceosome (ko03040), endocytosis (ko04144) and glycerophospholipid metabolism (ko00564) (**Figure S1A**). The metabolic pathways (ko01100) and the formation of secondary metabolites (ko01110) were linked DEGs in the MS1 vs. MS3 comparison (**Figure S1B**). Additionally, metabolic pathways (ko01100) and the manufacture of secondary metabolites (ko01110) were among the significantly enriched signaling pathways (**Figure S1C**).

### Statistics and functional enrichment of DEGs during the growth of female cone

**Figure 6:**
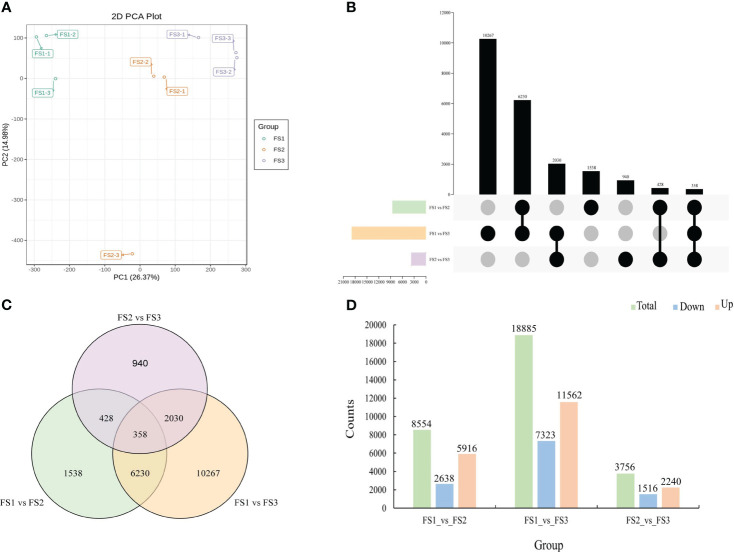
Identification of differentially expressed genes at the growth stages within female cone. **(A)** Principal component analysis (PCA) from different samples; **(B)** UpSet plot of DEGs in FS1 vs. FS2, FS1 vs. FS3 and FS2 vs. FS3; **(C)** Venn diagram of DEGs in FS1 vs. FS2, FS1 vs. FS3 and FS2 vs. FS3; **(D)** comparative analysis of upregulated and downregulated DEGs between the three comparison groups.

Gene expression patterns also varied dramatically during the growth of the female cone. A total of 498,720,034 raw reads from RNA-seq were collected, and 473,863,950 clean reads were obtained after screening and error correction. For all samples, the percentage change range for Q20 was 97.08% to 97.59%, with an average value of 97.45%. The average value was 92.92%, and the percentage of Q30 was higher than 92.27%. Additionally, the mapping rate of clean reads for each sample ranged from 84.76% to 85.67%, and the average GC content was 44.61%. These outcomes demonstrated the excellent level of RNA-seq data quality (**Table S4**). After that, DEseq2 was used to determine the gene expression levels in each group of female cone growth, and PCA results demonstrated that samples were accurately replicated at each stage (**Figure 6A**). All female cone growth genes together showed a total of 31,195 DEGs, including 18,885 DEGs (11,562 upregulated and 7323 downregulated) between FS1 and FS3, and 8554 DEGs (5916 upregulated and 2638 downregulated) between FS1 and FS2. Additionally, between FS2 and FS3, 2240 DEGs were upregulated, while 1516 DEGs were downregulated (**Figures 6B-D**).

Then, as shown in **Table S5**, Unigene annotation information was obtained by comparing Unigene sequences with a number of major databases. Genes associated with female cone growth were annotated in the GO and KEGG databases in proportions of 53.45% and 44.32%, respectively, and in the NR and KOG databases in multiples of 62.19% and 32.43%. **Figure S2 (A-C)** further compares the KEGG pathway analyses of DEGs among the three groups. The top four pathways in the FS1 vs. FS2 group were secondary metabolites (ko01110), plant-pathogen interaction (ko04626), MAPK signaling pathway-plant (ko04016), and plant hormone signal transduction (ko04075) (**Figure S2A**). Surprisingly, FS1 vs. FS3’s primary KEGG pathways resembled FS1 vs. FS2’s (**Figure S2B**). The most important pathways in FS2 vs. FS3 included metabolic pathways (ko01100), biosynthesis of various secondary metabolites-part 2 (ko00998), and MAPK signaling pathway-plant (ko04016) (**Figure S2C**).

### DEGs involved in plant hormone signal transduction and biosynthetic signaling pathways

**Figure 7:**
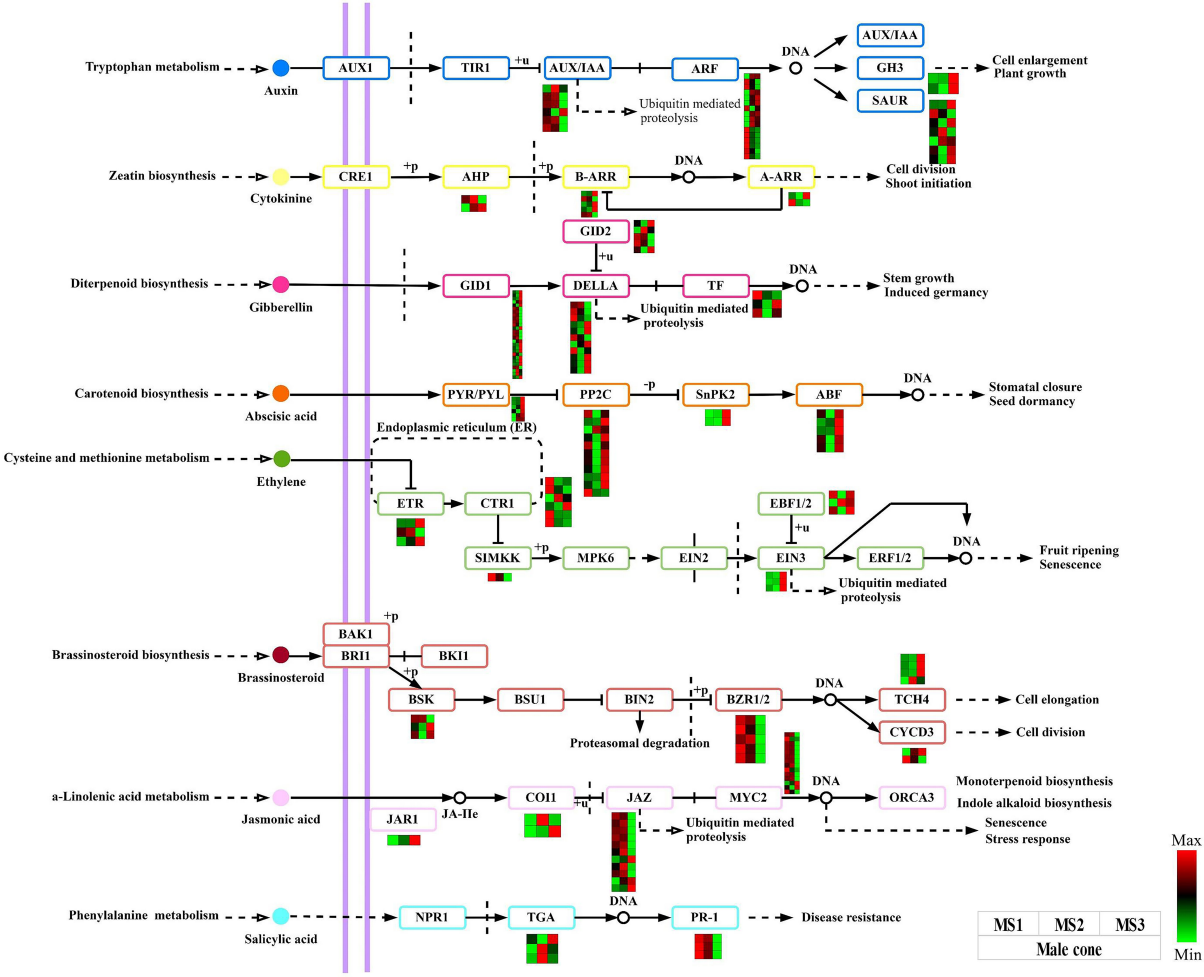
Analysis of DEGs related to the plant hormone signal transduction signaling pathway within male cone. The color scale of green to red refers to the min and max values, respectively.

We can draw the conclusion that the plant hormone signal transduction signaling pathway plays a crucial role in the growth of male and female cones based on the results of the DEGs detected and functional enrichment analysis. In this work, male and female cones, were shown to have 174 and 180 structural genes connected to this pathway respectively (**Tables S6**, **S7**). Heatmaps were created using the FPKM values of each gene (**Figure 7**, **Figure S3**). The IAA, CTK, GA, ABA, ETH, BR, JA, and salicylic acid (SA) pathways all had these genes active. The expression levels of 12 GID1 and 6 DELLA genes in the GA pathway of male cones were considerably greater in the MS3 stage than in the first two stages. The majority of the ABA pathway genes in male cones were also found to be strongly expressed at the MS3 stage, suggesting that these genes are essential for the late growth of male cones. The BZR1/2 and TCH4 genes were strongly expressed in the middle and late stages of growth in the BR pathway of female cones, suggesting that these genes have extensive biological activities in these phases of female cone growth.

**Figure 8:**
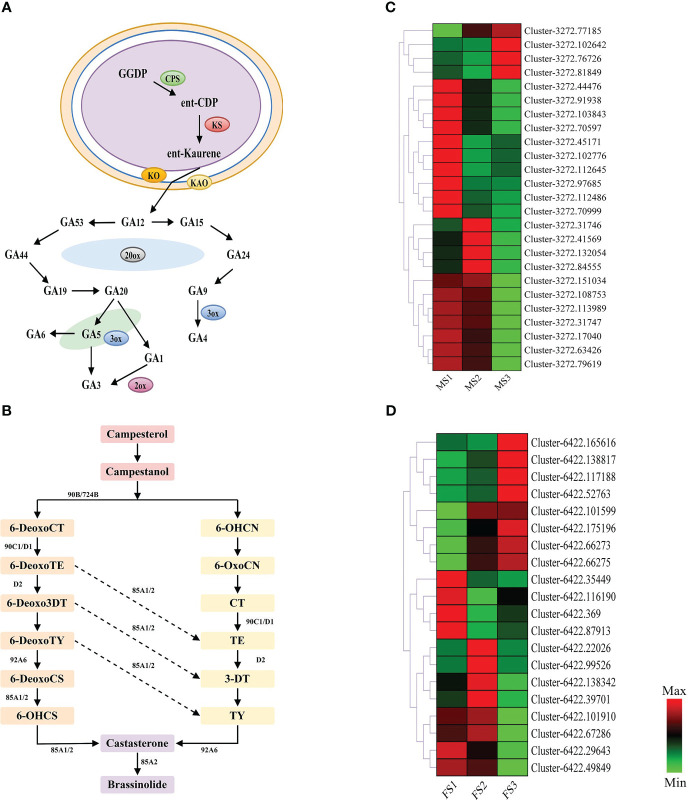
Genes related to gibberellin (GA) biosynthesis pathways in male cone, and brassinosteroid (BR) biosynthesis pathways in female cone. **(A)** GA biosynthesis pathways in male cone; **(B)** BR biosynthesis pathways in female cone; **(C, D)** heatmap cluster of the DEGs involved in GA and BR biosynthesis pathways. The color scale of green to red refers to the min and max values, respectively.

Furthermore, **Figure 4** reveals that GA and BR had higher concentrations at a later stage of growth in male and female cones, respectively. Therefore, this study annotated 25 and 20 EDGs to further identify the GA biosynthesis route in male cones and the BR biosynthesis pathway in female cones, respectively (**Figure 8**). These DEGs will improve the GA biosynthesis during the growth of male cones and BR biosynthesis during the growth of female cones.

### Statistical analysis of DEGs related to transcriptome factors and identification of MADS-box genes in male and female cones

**Figure 9:**
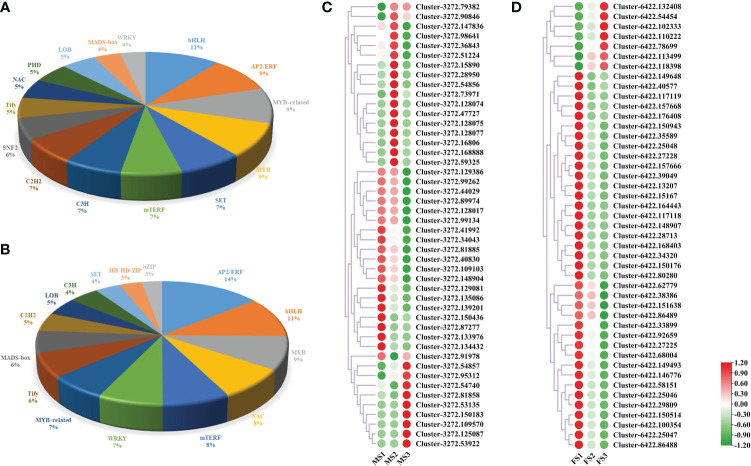
The DEGs involved in TFs. **(A)** TFs involved in male cone growth; **(B)** TFs involved in female cone growth. **(C, D)** Heatmap cluster of MADS-box DEG expression in male and female cones. Green and red circles represent MADS-box genes with low and high expression levels, respectively.

The time, location, and level of gene expression are all directly regulated by TFs, which are important molecules in this process (Cai et al., 2021). In this study, male and female cones were found to contain 2267 and 1227 genes related to these families of TFs, respectively. The top 15 TF families associated with the growth of male cones were bHLH (11, 5.69%), AP2/ERF (9, 5.07%), MYB-related (9, 5.09%), MYB (9, 4.72%), SET (7, 3.88%), mTERF (7, 3.66%), C3H (7, 3.57%), C2H2 (7, 3.48%), SNF2 (6, 3.00%), Tify (5, 2.87%), NAC (5, 2.78%), PHD (5, 2.78%), LOB (5, 2.65%), MADS-box (4, 2.03%), and WRKY (4, 1.94%) (**Figure 9A**, **Table S8**). Intriguingly, 13 of the top 15 TF families involved in the growth of female cones were identical to those in male cones, indicating that these TF families were very important in the growth of *P. koraiensis* (**Figure 9B**). Additionally, the expression of genes linked to the TF family of MADS-box during the growth of both male and female cones. The findings revealed that the discovered genes were expressed at various levels in the male cone’s MS1 to MS3 stamens, with a concentration in MS1 and MS2 (**Figure 9C**). However, the early growth of female cones was mostly influenced by genes from the MADS-box TF family (**Figure 9D**).

### Differential expression of DEGs related to cone flower growth development

**Figure 10:**
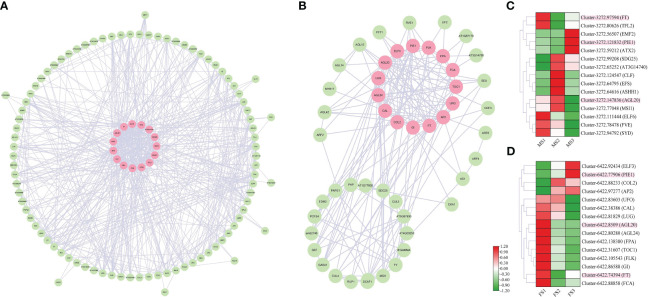
Coexpression network of identified DEGs in male and female cones growth development. **(A)** male cone coexpression network; **(B)** female cone coexpression network. **(C, D)** Heatmap cluster of DEG expression from the coexpression network in male and female cones. The genes with the strongest interaction with *A. thaliana* are marked in pink. Heatmap shows the expression level of DEGs involved in cone development, where the genes marked in pink are common homologues genes of both male and female cones.

The coexpression network was built using *A. thaliana* homologous proteins from the STRING database (http://string-db.org) to find the regulatory relationship between flowering-related genes with similar activities in the RNA-seq data of male and female cones. The coexpression network was viewed using Cytoscape software. We created the network in **Figure 10** using the *A. thaliana* protein database and the discovered genes associated with cone development. The strongest protein interactions between male and female cones and *A. thaliana* are represented by genes with pink labels (**Figure 10**). However, in the MS3 and FS3 stages, Cluster-3272.121832 and Cluster-6422.77906 were substantially expressed, indicating that they play a significant regulatory role in the later stage of cone flower growth development. It was discovered that many of the same *A. thaliana* protein genes were expressed in both male and female cones, indicating that these genes had comparable regulatory functions in the growth of both male and female cones.

### Quantitative real-time PCR assays of DEGs

**Figure 11:**
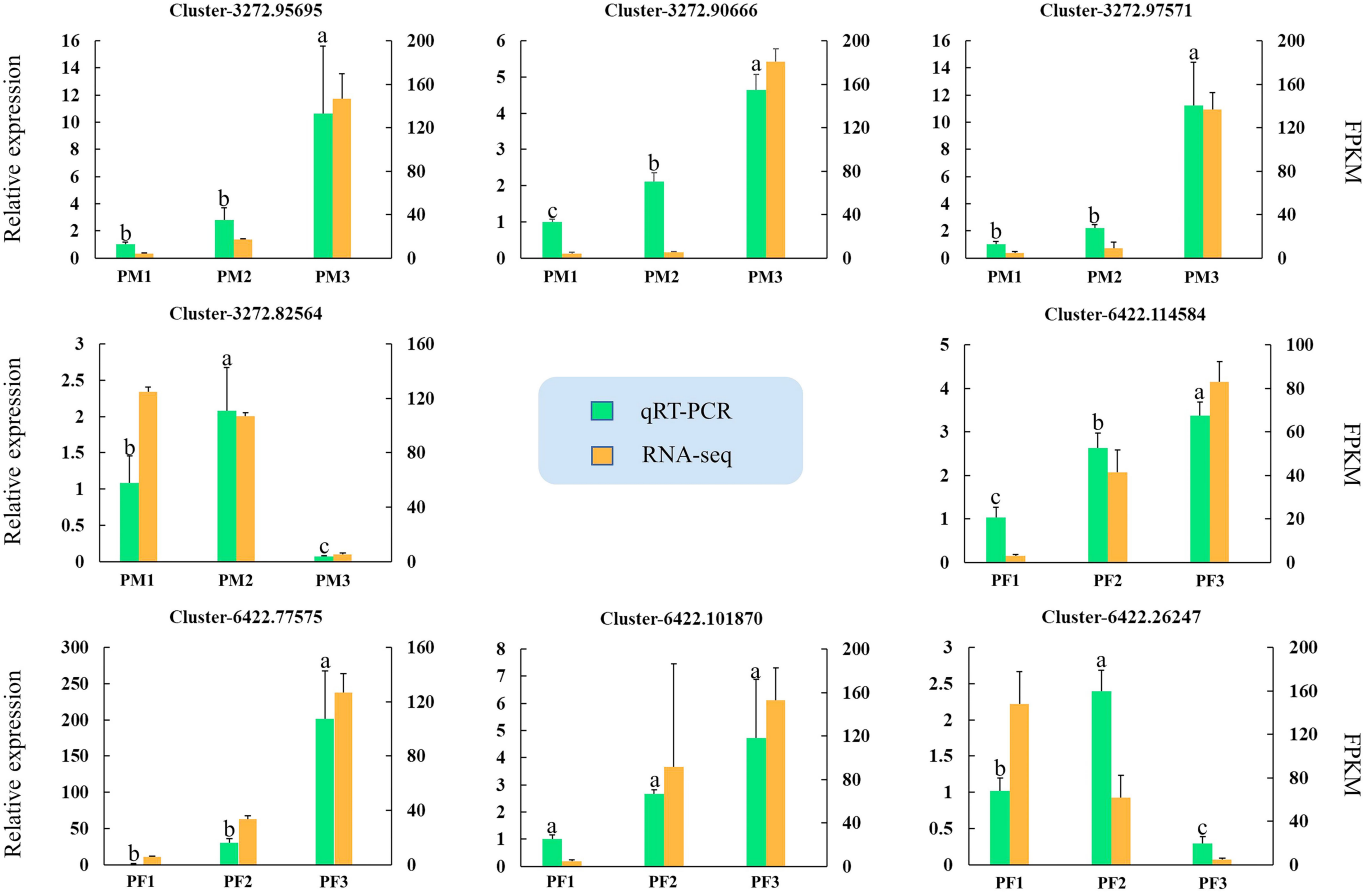
Quantitative real-time PCR assays of DEGs. The left y-axis represents the relative expression levels of genes by qRT-PCR, and the right y-axis represents the FPKM values of genes in RNA-seq data. The IBM SPSS Statistics v26.0 software with the Student-Newman-Keuls multiple range test was used to conduct the differences analysis, error bars represent the SD of the means at n=3, and bars with different lowercase letters are significantly different (*P*<0.05).

Eight potential genes from male (MS1, MS2 and MS3) and female (FS1, FS2 and FS3) cones were used for qRT-PCR verification to confirm the accuracy of the RNA-seq results. **Table S9** contains a list of the candidate gene primer sequences. **Figure 11** demonstrates that all candidate gene expression patterns were entirely compatible with the results of the RNA-seq study, suggesting the validity and reliability of the RNA-seq data.

## Discussion

The primary plant reproductive organ is the flower/cone. Its primary biological role is to make seeds by fusing female egg cells with sperm cells (Hoenicka et al., 2016; Dai et al., 2018; Diao et al., 2022). The flowering process is a crucial part of a plant’s whole life cycle and is primarily influenced by both internal physiological and external environmental influences. Early fruiting, high yield, and plant stability are all strongly correlated with the timing, quantity, and quality of flower growth development (Cerdan and Chory, 2003; Blackmore et al., 2007; Yang et al., 2010). *P. koraiensis* has significant commercial and ecological importance as a lumber and medicinal plant. However, *P. koraiensis* has a lengthy cone development period, a significant proportion of male cone, and a small proportion of female cone, and is therefore prone to lower yield. Additionally, some female cones will undergo abortion, which will lead to unsteady seed setting. Understanding the *P. koraiensis* cone flowering phase and molecular mechanism is crucial for increasing yield and production applications. To fill in the gaps in this crucial developmental process and lay a strong foundation for morphology and genetics research of *P. koraiensis*, the main process of male and female cone growth development of *P. koraiensis* was observed in this study. The molecular regulatory mechanism during cone growth was analyzed by transcriptome sequencing technology.

Understanding the process of cone growth development can be a guide for managing *P. koraiensis* production and artificial pollination at the right moment. In this study, we discovered that at the earliest stages of growth development, cone buds and leaf buds could not be separated. At 120 DAY, leaf buds were still extending with the rise in temperature, while cone buds were steadily thickening and displaying features. Gymnosperms such as *P. tabulaeformis* (Niu et al., 2016), *Pinus bungeana* (Liu, 1985), and *Pinus yunnanensis* (Chen and Wang, 1982) are widely known for taking two to three years from cone buds to cones. Additionally, a number of studies have noted that taxus plants are fertilized in the second year after pollination (Carlsbecker et al., 2013; Niu et al., 2022). The pollination period of *P. koraiensis* in this study was found to be between 160 and 165 DAY. When pollination was successful, the male cones withered while the female continued to grow until the second year, when the cones reached maturity. The study of the particular fertilization process, however, has not been documented, and this will be a critical scientific issue that must be resolved in the future. Furthermore, the link between cone buds and leaf buds is unknown, and distinguishing coniferous bundle cone buds has always been a contentious issue.

In response to certain environmental cues, plant cells actively manufacture chemicals known as phytohormones that can control physiological responses in plants. Phytohormones have significant regulatory effects on plant cell division and elongation, tissue and organ differentiation, blooming and fruiting, maturation and senescence, and dormancy and germination (Cheng et al., 2002; Ashikari et al., 2005; Kurakawa et al., 2007). All phases of flower development involve the synthesis of GA, which is crucial for flower growth development (Plackett et al., 2011; Bai and Xu, 2013; Song et al., 2013). The GA content in this study was significantly altered at various phases (*P*<0.05), demonstrating that GA is essential for the growth of *P. koraiensis* male cone. Additionally, the growth development of the male cone showed that the MS3 stage had a much greater GA content than the MS1 and MS2 stages, suggesting that gibberellin may play a more major role in the late stage of male cone growth development (**Figure 4**). Numerous investigations have demonstrated that BR is connected to flower growth development (Domagalska et al., 2007). For instance, Arabidopsis flowers later when the BR concentration is decreased (Shahnejat-Bushehri et al., 2016). The BR content was also significantly different (*P*<0.05) during the growth development of *P. koraiensis*’ female cones, and the accumulation was higher during the FS2 and FS3 phases, suggesting that it may played a key role in the later growth.

Based on morphological structure traits and physiological changes, the flowering process of plants is split into different stages. Currently, flowering determination, flower evocation and floral organ development are the three primary steps of a plant’s flowering process. According to earlier research, all flowering pathways are grouped together to form a regulatory network that interacts with one another to carry out each pathway’s specific regulatory function. The *CO*, *FLC*, *LFY*, *SOC1*, *FY* and *FT* genes are the key nodes of each regulatory convergence, among which the *LFY*, *SOC1*, *FY* and *FT* genes integrate the flowering pathway together (Komeda, 2004; Chen et al., 2022). The vernalization pathway has a significant impact on the timing of flowering in *A. thaliana*, and the genes *AGAMOUS LIKE19* (*AGL19*), *FLOWERING LOCUS T* (*FT*), and *FLC* are key players in this system (Suter et al., 2014). While *FCA* and *FY* work together to control *FLC* RNA processing, *FLC* is a MADS-box transcription factor that controls the activity of genes necessary for meristem development from vegetative development to floral development (Marquardt et al., 2006; Rataj and Simpson, 2014). In conifers, the function of *FT* gene is still controversial (Klintenas et al., 2012). Studies have showed that *FT* plays a key role in the formation and growth of Norway spruce bud set, but is not involved in the induction of *Pinus halepensis* cone flowering induction, but is related to the vegetative bud dormancy (Gyllenstrand et al., 2007; Reisfeld et al., 2022). In this study, transcriptome sequencing of male and female cones produced 71097 and 31195 DEGs, respectively, that were enriched in different cone flowering process pathways. In the protein coexpression network, we discovered *FT* (Cluster-3272.97594, Cluster-6422.74394) in the male and female cones. More evidence that these genes may have distinct regulatory effects on the early growth of male and female cones came from the discovery that these genes were significantly strongly expressed in the MS1 stage. One of the most important variables in regulating flower growth development is phytohormone signaling, which controls a variety of morphogenesis processes in plants (Davis, 2009). In higher plants, the biosynthesis of GA is mainly divided into three stages, and the decibel is catalyzed in the plastid, endoplasmic reticulum and cytoplasm (Eshed and Lippman, 2019; Wu et al., 2020). In this work, 25 DEGs were found to be active in GA biosynthesis during the growth of male cones, with high levels in the MS1 and MS2 phases that encouraged the buildup of gibberellin (**Figure 8A**). The GA signaling pathway’s negative regulator is the DELLA protein. The expression of associated genes that are either repressed or boosted by DELLA varies as the concentration rises, causing the protein to be destroyed through the ubiquitination pathway and facilitating plant flowering (Bao et al., 2020). In this study, 11 DELLA genes were discovered in male cones, which may promote the ubiquitination of the DELLA protein. The megaspore mother cell (MMC) is the first female germ cell differentiated from flowering plants. After developing into female gametophytes, it provides fertilization sites for plants. Studies have shown that BR biosynthesis and signal transduction genes accumulate in the sporophytes of ovule primordia. By activating WRKY23 transcription factors, BR signaling influences the growth of megaspore mother cells. The BRI1 receptor and BZR1 transcription factor family primarily control this activation (Cai et al., 2022). This study revealed the expression of six BZR1 genes in the female cone BR biosynthesis pathway, which may be crucial to the growth development of female cones.

This study lays a strong foundation for understanding the regulatory mechanism controlling the growth development of *P. koraiensis* cones, even though more research is still required to fully understand the process of flower bud transformation and sex differentiation prior to the development of *P. koraiensis* cones.

## Conclusions

In conclusion, after long-term phenological observation, we initially recorded the main developmental processes of male and female cones of *P. koraiensis*, and performed hormone content determination and transcriptome analysis on three crucial developmental stages. The results of hormone content determination showed that GA and BR were mainly accumulated in the late stage of development, indicating that they may be the key hormones to promote the development and maturation of male and female cones. Then, a total of 71,097 and 31,195 DEGs were identified in this research, which provided available genetic information for the study of the molecular breeding of *P. koraiensis*. In addition, this study focused on the hormone signal transduction pathway and biosynthesis pathway during the development of male and female cones, and evaluated and discussed them. Combined with transcriptome data, all transcription factors were screened out, and the expression levels of important genes MADS-box related to flowering were analyzed. Finally, the coexpression network was used to further predict that Cluster-3272.121832 and Cluster-6422.77906 may be the crucial regulatory genes for the development of male and female cones of *P. koraiensis*.

## Data availability statement

The datasets presented in this study can be found in online repositories. The names of the repository/repositories and accession number(s) can be found in the article/**Supplementary Material**.

## Author contributions

YL: Conceptualization, Data curation, Formal Analysis, Investigation, Methodology, Writing – original draft, Writing – review & editing. MZ: Methodology, Writing – review & editing. KC: Methodology, Writing – review & editing. LL: Software, Writing – review & editing. RH: Software, Writing – review & editing. XP: Data curation, Writing – review & editing. LZ: Data curation, Writing – review & editing. XZ: Data curation, Funding acquisition, Resources, Validation, Writing – review & editing.
