# Supplementary material for: Phytohormone biosynthesis and transcriptional analyses provide insight into the main growth stage of male and female cones Pinus koraiensis
Source: Front Plant Sci. 2023 Oct 11;14:1273409. doi: 10.3389/fpls.2023.1273409 (PMC10598626; doi:10.3389/fpls.2023.1273409)
Supplement: Supplementary file 1 [file DataSheet_1.docx]

Supplementary Material

**Phytohormone biosynthesis and transcriptional analyses provide insight into the main growth stage of male and female cones *Pinus koraiensis***

**Yan Li^1,2^, Minghui Zhao^1^, Kewei Cai^1^, Lin Liu^1^, Rui Han^1^, Xiaona Pei^3^, Lina Zhang^4*^, Xiyang Zhao^1*^**

*** Correspondence:** Lina Zhang [zhangln@jlau.edu.cn](mailto:zhangln@jlau.edu.cn) XiyangZhao [zhaoxyphd@163.com](mailto:zhaoxyphd@163.com)

# Supplementary Figures and Tables

## Supplementary Figures


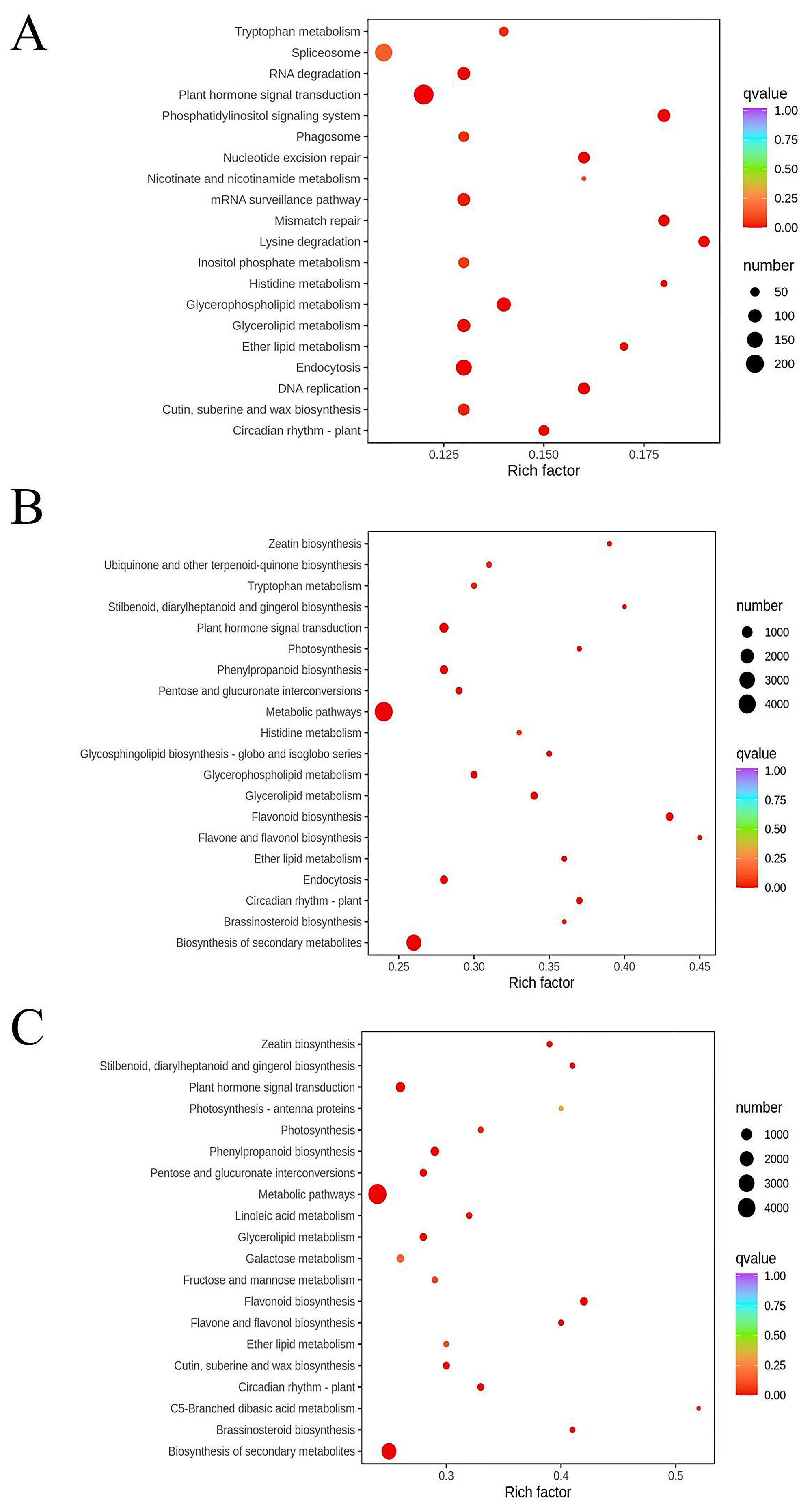


**Supplementary Figure 1.** The KEGG enrichment analysis of differentially expressed genes during male cone development. (A-C) suggest the top 20 enriched KEGG terms of the DEGs in MS1 vs. MS2, MS1 vs. MS3 and MS2 VS. MS3, respectively.


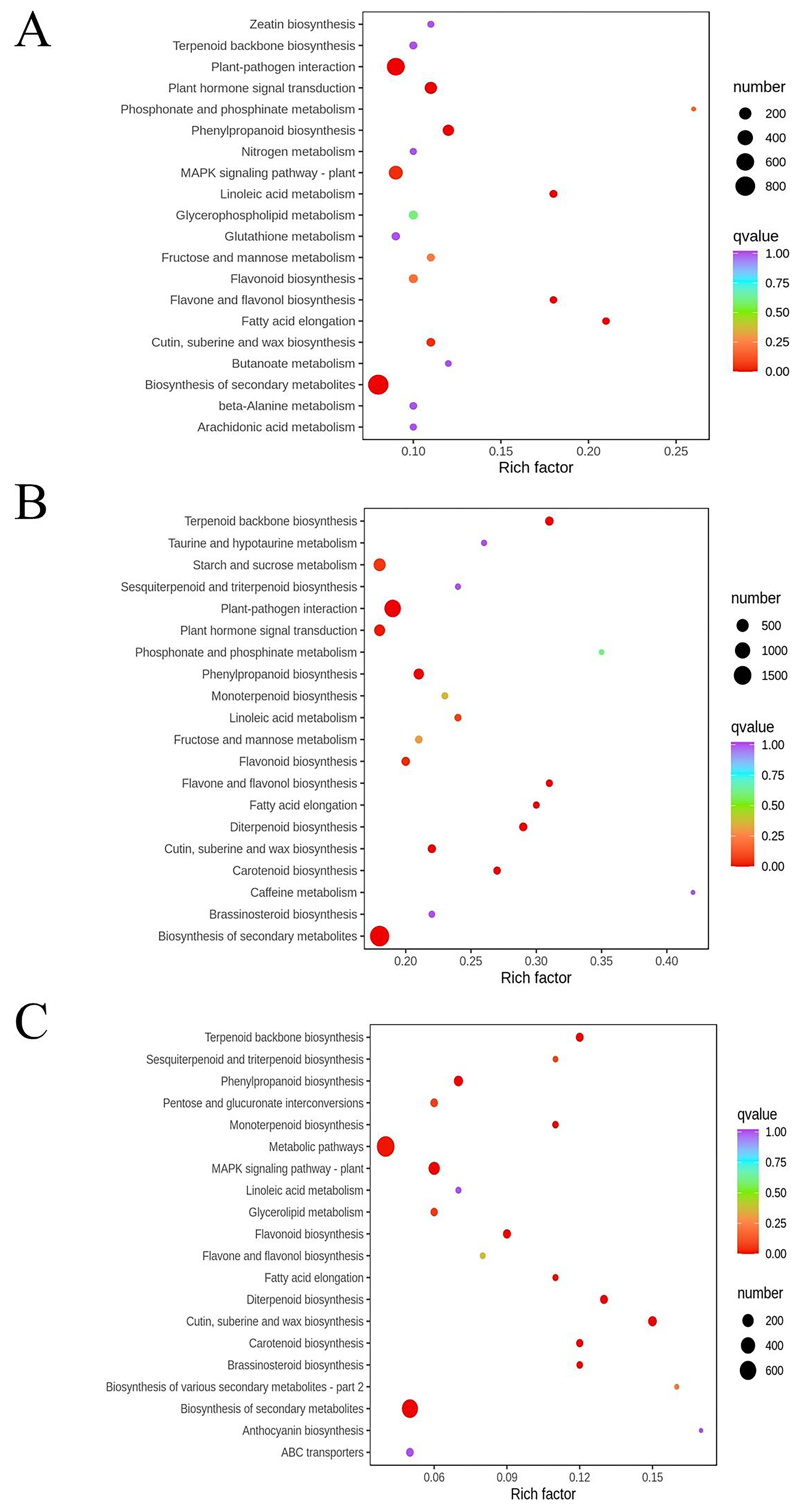


**Supplementary Figure 2.** The KEGG enrichment analysis of differentially expressed genes during female cone development. (A-C) suggest the top 20 enriched KEGG terms of the DEGs in FS1 vs. FS2, FS1 vs. FS3 and FS2 VS. FS3, respectively.


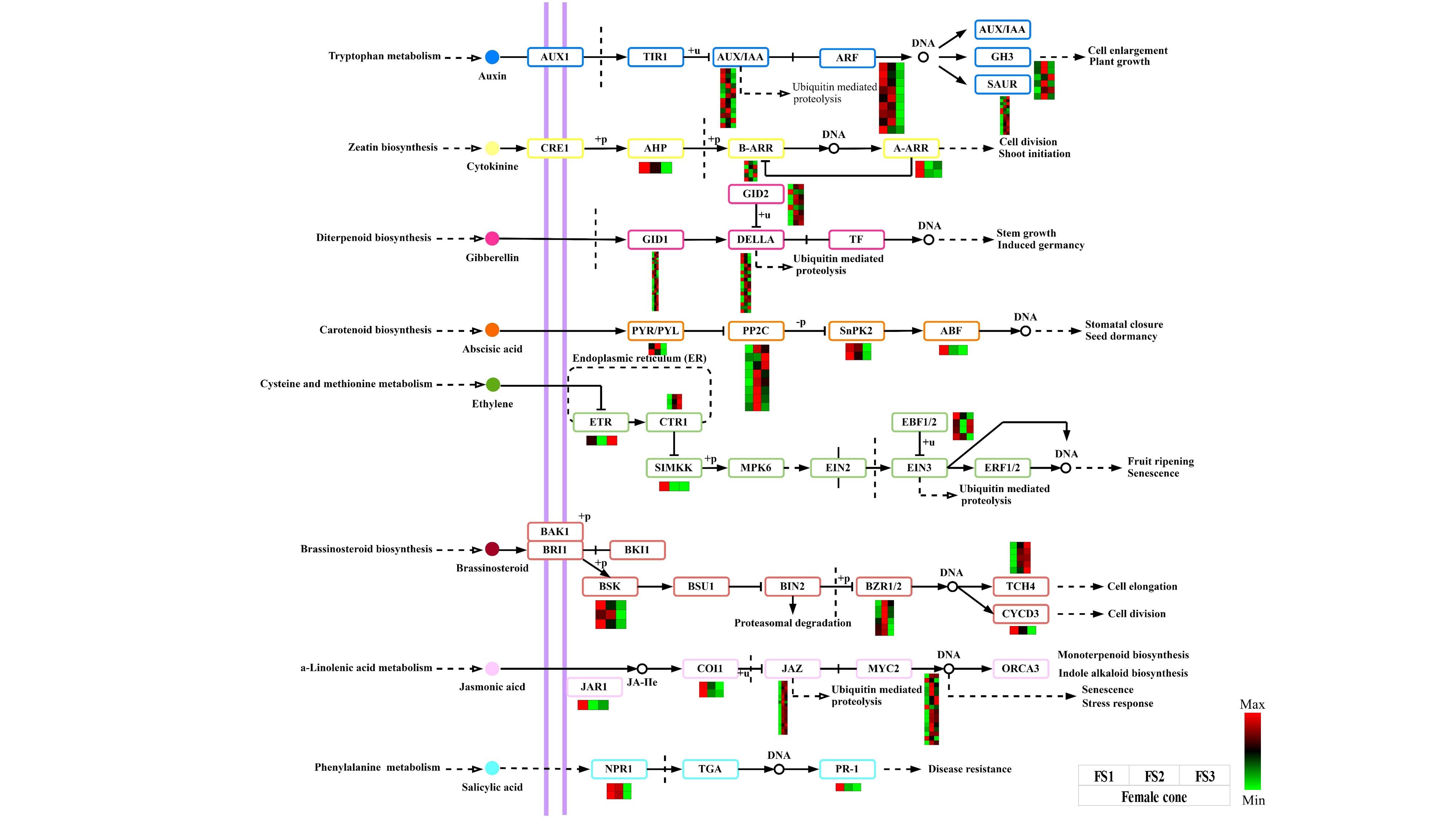


**Supplementary Figure 3.** Analysis of DEGs related to the plant hormone signal transduction and biosynthetic signaling pathway within female cone. Color scale of green to red indicates to the min and max value, respectively.
